# Medical Opinion and Movement

**Published:** 1911-08-26

**Authors:** 


### Medical Opinion and Movement.

### Treatment of Furunculosis by Collodion.

For some years past M. Fuchs, as described in the *Münch. Med. Woch.*, has treated furunculosis by means of a ring of collodion applied round the inflamed spot. The material is painted on with an ordinary camel's hair brush in such a way as to surround the furuncle while leaving its central portion uncovered. The extent of the uncovered area varies with the size of the furuncle, and in any case it should not be reduced too much, but should be at least of a size that would need a sixpenny-piece to cover it. The collodion ring must be renewed several times a day by passing the brush carefully over the area already covered and leaving the centre untouched. This last comes to stand out more and more, whereas the collodion ring tends to encircle it more closely. The peripheral inflammation is the first to disappear, and next the central process gradually becomes less acute; in a day or two a yellow point appears, which bursts and extrudes the core, after which cure is complete. The peripheral constriction caused by the encircling collodion is the means whereby the inflammation is reduced, and the substance acts as a protective to prevent further extension of the inflammatory process.

### The Ætiology of Endemic Goitre.

The researches of Captain M'Carrison into the peculiar goître prevalent on the Indian frontier about Gilgit have been noticed on a previous occasion in these columns. In his latest communication on the subject this author states that there exists in suspension in waters which are known to be goître-producing an agent which is capable of initiating an hypertrophy of the thyroid gland. This agent is destroyed by boiling, and is removed from the water by filtration; it is therefore either a living organism or a chemical substance the noxious properties of which are destroyed by heat. The incubation period of experimentally produced goître is usually about ten to fifteen days. This goître can be cured by the administration of intestinal antiseptics; lactic ferments exercise a curative action when applied to the treatment of incipient goîtres. It is probable that the agent responsible for the production of goître is a living organism parasitic in the human intestine. The disease cannot be communicated to dogs by means of watery extracts from the fæces of goîtrous individuals. How far these deductions as to the endemic goîtres seen in Chitral are applicable to sporadic goître as it occurs in Europe or to the endemic disease of Derbyshire, Switzerland, etc., is a wider question; but the possibility seems worth bearing in mind by anyone looking out for a chance of doing research work.

### The Transmission of Cretinism.

Dr. Lemierre, in the *Gazette des Hôpitaux*, reports the conclusions arrived at from the studies of Von Aichbergen on the transmission of cretinism from man to animals. This author has for many years made a study of cretinism in Styria, and is convinced that the affection must be ranked among the infectious diseases. He had the opportunity of observing two dogs brought up by a female "demi-cretin," who made them sleep in her own bed. In course of time the dogs developed cretinous characteristics. Badly developed, they presented voluminous goîtres, their coats were dull, dry, and lanuginous, and they retained their milk teeth. They were unable to bark and appeared devoid of all intelligence. The author next entrusted this woman with a young dog obtained from an absolutely healthy litter, without hereditary pathological taint. The young animal took up the same style of living as the two others removed from the house. Three months later it also had a goître, but was still lively and able to bark. Ten months later the goître had become enormous, the animal was apathetic and incapable of barking. Its coat had lost its gloss, and its milk teeth remained. In short, it presented the appearance of a cretin. On the other hand, a dog of larger breed entrusted to the woman and brought up in the same room as its companion, but without sleeping in the same bed, developed on normal lines. The author thinks that these facts demonstrate that the transmission of cretinism from man to animals is possible.

### The Dilatation of Strictures.

In the *Northumberland and Durham Medical Journal* Mr. R. J. Willan lays down the following rules for the management of strictures of the urethra. Every patient who has had gonorrhœa should be examined periodically for five years after his infection, best with the urethroscope. A gonorrhœal stricture is nearly always multiple; treatment likely to increase the amount of fibrous tissue must be avoided. Intermittent dilatation with flexible instruments not oftener than twice a week is the ideal treatment: flexible instruments are always preferable to solid ones (except in a wide-calibred stricture), as they produce less trauma. In continuous dilatation a flexible instrument only should be used, and then only when there is acute retention; a solid instrument should not be tied into the bladder. Rapid dilatation is bad; it is better to do internal urethrotomy. After a stricture has been dilated to full size, a large bougie should be passed annually for some years.

### The Symptoms of Round Worms.

*Ascaris lumbricoides* cannot be called uncommon in Britain; but in some countries it is practically universal, and Dr. J. P. Maxwell, writing in the *China Medical Journal*, summarises a very extensive experience. He is quite sure that opium smokers suffer less than ordinary individuals from this infection, and that when they are infected they harbour smaller numbers of the parasites. Quinine and chloroform are two more drugs whose exhibition seriously prejudices the well-being of a colony of round worms. As regards signs and symptoms, he attaches value to the following: A craving for food about an hour after a good meal; not exactly a sensation of pain, but at least one of an unpleasant character. A large flabby protuberant abdomen in a child, looking as if it might be due to enlarged spleen, should excite suspicion. Discomfort in the stomach unconnected with food may indicate the presence of worms in that viscus; and sometimes patients can even feel the movements of the parasites there. In such cases santonin has produced immediate vomiting of *ascarides*. Perversions of appetite, anorexia, voracity are also seen; sometimes infected children pick plaster off walls to eat, or have a craving for earth. In children diarrhœa, or even dysenteric stools, may be set up by the irritation of the worms. Occult blood is practically never found in the stools when tested for. Anæmia with eosinophilæmia may occur, but neither is at all constant.

### Infantile Eczema and High Altitudes.

At a meeting of the Académie de Médecine, Professor Marfan gave an interesting account of his treatment of severe eczema in infants by removing them for some weeks to a place of high altitude. This method of treatment is, of course, only necessary in severe cases in which the eczematous lesions are extensive and associated with general symptoms of a serious nature—malnutrition, loss of weight, fever, insomnia, and cachexia. Such cases, as the author points out, are extremely rebellious to ordinary methods of treatment and fail to yield to the most careful regulation of diet and general and local medication. In these cases the author has obtained most successful results by ordering a sojourn for a month to six weeks to some place 1,000 to 1,500 metres above the sea-level. His observations extend altogether over fifteen cases. Of these, the cure only failed in one case, and in another case the condition improved, but the baby was brought back at the end of a week as it did not sleep. In the remaining thirteen cases the results were entirely satisfactory. For the first few days there was sometimes an exacerbation of the condition, but in the following week improvement set in, the eruption began to clear up, the pruritus diminished, and sleep became normal. By the end of a fortnight there was usually only a little dry eczema remaining, and the child began to put on weight. In three cases the cure was permanent; in seven cases there was some return of the trouble afterwards, but never in the same intensity. In three cases finally there were further attacks of considerable severity after return from the mountains, but they soon yielded to ordinary treatment and did not seriously affect the general health. The author admits that improvement often follows a change of air, either to the country or seaside, but is of opinion that a much more beneficial effect is obtained by a sojourn at a high altitude, and suggests that there is something specific in the mountain conditions, whether it be the rarefaction of the air, the quality of the light, the atmospheric electricity, or some other factor of which we are at present ignorant.

### Meningeal Typhoid.

An interesting case of meningeal typhoid is recorded by Dr. Stühmer, of Magdeburg, in the *Münchener Medizinische Wochenschrift.* The patient was a young man admitted into hospital for general malaise of several days' duration. He vomited once on the day of admission, but otherwise there were no abdominal symptoms. The abdominal wall was slightly distended, but not at all sensitive on palpation, and there were no spots and no enlargement of the spleen. Bronchial râles were detected in the chest. The patient was very agitated and nervous, and was delirious at night. Two days after admission rigidity of the neck was noticed, which increased. The pupils reacted sluggishly. The fundi were normal. The Widal reaction was positive for 1.50, negative for 1.100. As the meningeal signs became more marked, lumbar puncture was performed. The cerebro-spinal fluid escaped under great pressure, and microscopic examination revealed the presence of Eberth's bacilli. The symptoms rapidly abated after the puncture, the temperature fell from 39.8° C. to normal, and the patient was soon well. Although such a case is sufficiently rare, the author has been able to collect eight similar cases in medical literature, in which the typhoid bacilli have been detected in the cerebro-spinal fluid. In all these cases the cardinal typhoid symptoms were absent. Five of them recovered. In such cases lumbar puncture is of the greatest value, not only as a means of diagnosis but also as a therapeutic measure.

### A Vaccine for Leprosy.

Major Rost reports very fully from Rangoon on twelve cases of leprosy treated by him with a vaccine prepared from cultivations of the leprosy streptothrix. The reports are given in great detail, and it appears that five of the twelve patients are now cured as far as clinical observation goes; all the other seven are remarkably improved, and some of them are nearly cured. Condensing his experience of this treatment, he says that in nodular cases only a slight reaction should be aimed at; whereas in anæsthetic cases, the greater the reaction the better the result. The reason of this is probably that in the anæsthetic cases the bacilli are actually in the nerves, and thus there is less danger of metastasis than there is in the large masses of the nodular form. Lastly, it must be noted that the treatment needs perseverance; the injections are given once a week for long periods, and in some cases the benefit has appeared only after months of treatment. The author is starting to treat several fresh cases, clinical reports of which he proposes to submit at a later date. The present article is in the *Indian Medical Gazette.*

### The Diagnosis of Dementia Præcox.

In 1909 it was asserted by Much and Holzmann that the serum of patients suffering from dementia præcox and maniacal depressive insanity is able to inhibit the hæmolysis of normal red blood cells by cobra venom. Normal individuals and patients with other kinds of insanity were said not to exhibit the same reaction. This test was tried by many other observers, chiefly German, who failed for the most part to confirm the absolute precision claimed for it by its authors. Some investigators agreed that the reaction is always found in these two forms of insanity, but denied that it does not occur in other forms of insanity; and some could not even agree that it always occurs in the two specified forms of insanity. In the *Johns Hopkins Hospital Bulletin* Drs. Moss and Barnes record the results of their researches upon forty-nine individuals, ten of whom were sane and the rest not sane. Many of the patients were repeatedly tested, but without reference to their psychic state at the moment. There were twenty-one cases of dementia præcox and four of maniacal depressive insanity among the thirty-nine insane tested. As a result the authors concur in the view that the Much-Holzmann reaction has no diagnostic significance. They also find that corpuscles from normal individuals vary in their resistance to cobra venom to a slight extent, and also that the action of different sera in favouring or retarding cobra venom hæmolysis is inconstant.

### The Bacillus of Scarlatina.

On several occasions bacteriologists have announced the discovery of the organism of scarlet fever, usually a coccus of some kind. Hitherto each of these enthusiasts has been proved to be mistaken; and some indeed have argued that the particulate contagion is too small to be seen microscopically, since it is believed to be capable of passing through a Berkefeld filter. Dr. Vipard, of Montreal, announces in the *Archives of Pediatrics* that the prime cause is really a bacillus, which he has isolated from the lymphatic glands of seven cases of scarlet fever. It grows rapidly on all ordinary media and is an active spore-former. The bacilli thus cultivated have been injected into five monkeys and two rabbits, all of which developed in from two to five days an acute illness characterised by rash, enlarged glands, and desquamation. The enlarged glands of these animals yielded the same bacillus in pure culture. Moreover, the patients thus inoculated have infected other non-inoculated monkeys and rabbits by direct contagion, and from the inguinal glands of these secondarily infected animals the same bacillus has been once more recovered. The bacilli thus recovered from infected animals have exactly the same cultural characteristics as those from the original human cases. The author expressly states that this is a preliminary report only, and that he hopes presently to present a much larger series of observations.

### Vaccines in Acne.

The vaccine treatment of acne vulgaris used at one time to be chiefly by means of anti-staphylococcal vaccine; the cause of the failure of this in many cases is that the essential micro-organism of acne itself is the bacillus acnes, the staphylococcus albus being a secondary organism that produces suppuration in the primary comedo. Dr. G. T. Western, in the *British Journal of Dermatology*, vol. xxii., No. 1, p. 6, summarises modern views upon the subject, and divides cases up into three main groups: (1) Those in which the comedo is the dominant feature; (2) Those in which induration is most marked (acne indurata); and (3) Those in which the outstanding feature is abscess formation (acne pustulosa). The specific treatment of the first group is by means of a vaccine prepared from the bacillus acnes; in the second group the specific treatment requires a vaccine prepared both from the bacillus acnes and from the staphylococcus albus, whilst in the third group vaccines prepared from the staphylococcus albus are particularly indicated. He gives illustrative cases and points out that the dosage of the acne vaccine should be very small compared with that of other vaccines; products of from three to fifteen million being sufficient for a dose in most cases.

### The Latest Treatment for Tuberculosis.

In *La Gazette Médicale Belge* Drs. Samuel Bernheim and Louis Dieupart give a résumé of the work of Drs. Szendeffy and Kertéz Aba, of Budapest, as well as that of other observers, with radio-active iodo-menthol in the treatment of tuberculosis. This, the latest treatment for consumption, has already been noticed in The Hospital, when adverse comment was made upon the fact that a series of advertisements had appeared with reference to it in one of the great London dailies. The Belgian observers cite seventy-five cases treated by themselves and others with the new method, including one of tuberculous epididymitis, several of tuberculous adenitis and laryngeal tuberculosis, four of tubercle of bone, and a large number of cases of pulmonary tuberculosis. They report a certain proportion of definite cures, together with a number of cases in which marked improvement nearly approached a cure. They state that the drug is a powerful dynamogenic agent. Injected by the intramuscular route it destroys the associated micro-organisms, such as the streptococcus, staphylococcus, and pneumococcus, and succeeds in so altering Koch's bacillus as to make it degenerate, to colour badly, and finally to disappear altogether. Parallel with this disappearance of the pathogenic elements the morbid symptoms grow less marked and in the end clear up. Intramuscular injections are painless and present no reaction, even in children. Daily doses to the number of thirty are given in this way, after which the treatment is discontinued for a fortnight. The drug is very active and its effects are produced from the moment it is first administered. From two to four series of injections are generally necessary to ensure a definite cure. It goes without saying that the treatment is only applicable in the case of patients whose general health is still satisfactory and not in the case of cachectics. For our part we should prefer to suspend judgment with regard to this form of treatment of tuberculosis until as many hundreds of patients have been subjected to its influence as there have up to the present been single persons.

